# Epidemiological insights into colorectal cancer in northwestern Algeria

**DOI:** 10.3332/ecancer.2024.1684

**Published:** 2024-03-21

**Authors:** Salah Eddine El Herrag, Soraya Moulessehoul, Douniazad El Mehadji, Djamila Yekrou, Méghit Boumediène Khaled

**Affiliations:** 1 Laboratoire de Nutrition, Pathologie, Agro-Biotechnologie et Santé (Lab-NuPABS), Faculty of Natural and Life Sciences, Djillali Liabes University, Sidi Bel Abbes 22000, Algeria; 2 Department of Biology Rectorat Ex ITMA, Faculty of Natural and Life Sciences, Djillali Liabes University, BP 89, Sidi Bel Abbes 22000, Algeria; 3 Department of Medical Oncology, Anti-Cancer Centre of Sidi Bel Abbes, Sidi Bel Abbes 22000, Algeria; a https://orcid.org/0000-0002-6483-4771; b https://orcid.org/0000-0002-0214-6383; c https://orcid.org/0000-0001-5281-2498

**Keywords:** colorectal neoplasms, epidemiology, incidence, Algeria

## Abstract

**Objectives:**

The incidence of colorectal cancer (CRC) has exhibited regional variability in North Africa and the Middle East, with a steady increase in Algeria. Despite this trend, limited data exist on the epidemiology of CRC in northwestern Algeria. Our study aimed to investigate the epidemiological characteristics of CRC in this region.

**Methods:**

We conducted a retrospective study examining 255 confirmed CRC cases through medical records from patients at the Sidi Bel Abbes anti-cancer centre.

**Results:**

The mean age of the study participants was 59 ± 13 years. The results showed a higher incidence in males (57%) than in females, and colon (62%) than rectal cancer. Within this cohort, 47% had a pre-existing medical condition, while 39% had a family history of cancer. Adenocarcinomas were the prevailing histological subtype in 94% of CRC cases. Compared with colon cancer, rectal cancer was less often diagnosed at stage IV of the disease (OR = 0.75; 95% CI = 0.09, 4.86; *p* = 0.8) and more likely in early-onset patients (OR = 2.27; 95% CI = 1.25, 4.17; *p* = 0.007). Men were at a higher risk of being diagnosed with metastatic CRC primarily hepatic metastases (OR = 2.03; 95% CI = 1.07, 3.99; *p* = 0.033) and pulmonary metastases (OR = 2.50; 95% CI = 1.07, 6.59; *p* = 0.045).

**Conclusion:**

This study may provide a comprehensive glimpse into CRC epidemiology in northwest Algeria. Understanding regional differences is the key to implementing specific preventive and interventional strategies.

## Introduction

Colorectal cancer (CRC) is a major global health concern with varying incidence rates among populations worldwide. In Africa, the age-standardised rate per 100,000, was 8.4 [1], which is generally lower than that in other parts of the world, with notable heterogeneity from regional and country-level perspectives [2]. Data from the Middle East and North Africa show a comparatively rising trend [3]. CRC has emerged as a growing health concern in Algeria owing to the severity of the disease, as its incidence rate continues to rise. The number of new CRC cases has significantly increased in recent years. In 2020, GLOBOCAN reported that the number of new CRC cases identified in Algeria nearly doubled from the previous year, marking a 94% increase in new cases. This alarming trend has allowed CRC to rank second in terms of cancer incidence in Algeria after breast cancer and to have the third highest mortality rate after lung and breast cancer in both sexes and all age categories [1, 4].

CRC is a complex and multifactorial neoplasm that is influenced by a variety of factors, including genetics, environment and lifestyle [5–8]. Previous studies have shown that the incidence, prevalence and clinical characteristics of CRC vary depending on geographic and demographic factors [3, 9–11].

Data on CRC burden in the northwestern region of Algeria remain scarce. On this basis, we have attempted through the present investigation to shed light on some of the demographic, epidemiological, clinical, histological and therapeutic aspects of CRC patients in the region. This study will contribute to expanding knowledge on the epidemiology of CRC globally with data from the Algerian population and, specifically, the province of Sidi Bel Abbes, located in the northwest of the country.

This study aimed to assess CRC’s epidemiological profile in Algeria’s western region, which can provide new insights into the incidence and risk factors for CRC in this population.

## Methods

### Study design and setting

We performed a retrospective cohort investigation involving patients with histologically confirmed CRC identified from patient records admitted to the anti-cancer centre of the University Hospital of Sidi Bel Abbes, Algeria. The reporting of the study adhered to the guidelines outlined in the strengthening the reporting of observational studies in epidemiology checklist.

The anti-cancer centre is a regional health facility designed for the care of patients with cancer in the western and southwestern regions of the country. The study spans 4 years (January 2018 to December 2021).

The study was approved by the Biology Department Committee of the Natural and Life Sciences Faculty of Djillali Liabes University and the Department of Medical Oncology of the Anti-cancer Centre of Sidi Bel Abbes. The ethics committee of University Hospital Centre Abdelkader Hassani of Sidi bel Abbes approved the study (Ref: Decision N^o^ 5).

### Study participants

Subjects were identified through paper-based medical records of patients admitted to the centre with a confirmed diagnosis of a primary CRC tumour. All patients meeting the eligibility criteria i.e., presenting a histologically confirmed primary CRC, were included in the final analysis without applying any exclusion criteria.

### Variables

#### Demographics

Data on age, sex, anatomic site, socioeconomic status, higher educational status, height and weight were collected directly from the patient’s medical records. Patients were categorised into two groups: those with early-onset CRC (diagnosed before the age of 50 years) and those with late-onset CRC (diagnosed at 50 years of age or older). Body mass index (BMI) was calculated by dividing the patient’s weight (in kg) by the square of their height (in m). Subjects were subsequently categorised based on their BMI into distinct groups: underweight (BMI ≤18.4 kg/m^2^), normal weight (BMI 18.5–24.9 kg/m^2^), overweight (BMI 25.0–39.9 kg/m^2^), and obese (BMI ≥ 40.0 kg/m^2^), in accordance with the criteria established by the centres for disease control and prevention [12]. Patients were stratified according to anatomical site (colon or rectum C18-C20) [13].

#### Personal medical, surgical and family history of cancer

We extracted information related to the personal medical, surgical, and cancer family history of all patients included in the study. First-degree relatives included mothers, fathers, daughters, sons, sisters and brothers. On the other hand, individuals categorised as second-degree relatives encompass progenitors and descendants spanning multiple generations as well as collateral relatives such as paternal and maternal aunts, uncles, nieces, nephews, grandparents and grandchildren. Other personal medical history encompasses comorbidities beyond hypertension, diabetes and stroke, while other personal surgical history includes surgical interventions apart from appendicectomy or cholecystectomy.

#### Histological classification, staging and metastasis

We collected information on the tumour histological classification, staging, and metastasis status. The American Joint Committee on Cancer cancer staging system, specifically the tumour, node and metastasis scoring algorithm, is employed for CRC staging [14].

#### Treatment and symptoms

The centre provides treatment for CRC patients including chemotherapy, radiotherapy and targeted therapy. Symptoms at diagnosis were retrieved from the medical records.

#### Lifestyle factors and biomarkers

Data collected included information on biomarkers, including carcinoembryonic antigen (CEA) and carbohydrate antigen 19-9, also denoted as cancer antigen 19-9 or sialyl Lewis A antigen (CA 19-9) levels, as well as the mutational status of Kirsten rat sarcoma virus (KRAS), Neuroblastoma RAS (NRAS) and B-Raf Proto-Oncogene, Serine/Threonine Kinase (BRAF). These biomarkers are acknowledged for their potential to aid in CRC diagnosis, prognosis and management [15, 16]. Additionally, patients’ records also contained data on smoking and alcohol consumption, providing valuable information for assessing potential risk factors and their influence on the disease.

### Statistical methods

Numerical variables were summarised using means (measures of central tendency), standard deviations, and ranges (measures of dispersion). Categorical variables, on the other hand, were summarised using frequencies and percentages from the total number of observations, and missing data were not accounted for in percentage calculations. The Wilcoxon rank-sum test was employed to examine the differences between two groups of continuous distribution.

Pearson’s chi-squared test was applied to ascertain the significance of the association between categories. Alternatively, Fisher’s exact test was utilised to ascertain the significance of the association between two categories in the case of a small sample size. The logistic regression model using maximum likelihood estimation was used to predict the probability of a binary outcome and calculate the odds ratios (OR) and their 95% confidence intervals (CI) to assess potential correlations between age (early-onset versus late-onset), sex (male versus female), and anatomic site (colon versus rectum) and patients’ characteristics. The results of all statistical tests were deemed significant at a *p*-value of <0.05 threshold. All statistical analyses were performed using the *R* statistical program (version 4.3.1) [17, 18].

## Results

### Participants

Our study included 255 confirmed CRC patients diagnosed at the level of the anti-cancer centre of Sidi Bel Abbes, located in the northwestern region of Algeria.

### Descriptive data

57% of patients were male (*n* = 145), and 43% were female (*n* = 110). Colon cancer was the most frequent anatomical site, accounting for 62% (*n* = 158) of all CRC cases. The mean age of the study population was 59 ± 13 years (range, 20–91 years); 93% were 40 years or older, and 78% were diagnosed at 50 years of age or more. Female patients were slightly younger than their male counterparts, with a mean age of 58 ± 13 years (range, 22–89 years) and 60 ± 13 years (range, 20–91 years) (*p* = 0.300) for males (Table 1). We noticed a significant difference in the mean age at diagnosis according to the CRC anatomical site (mean age = 61 ± 13 for colon cancer versus 57 ± 13 for rectum, *p* = 0.011). Patients aged 50 years or older demonstrated a higher likelihood of being diagnosed with colon cancer than rectal cancer (OR = 2.27; 95% CI = 1.25, 4.17; *p* = 0.007).

Despite the limited available data, significant sex-based disparities were observed where female patients were more likely to have a low socioeconomic level (*p* = 0.031) and less likely to have a higher educational status (*p* = 0.014) compared to male patients.

The mean height of the overall population was 1.66 ± 0.10 m, and the average weight was 63 ± 13 kg. Male patients were taller (mean height = 1.71 ± 0.08 versus 1.58 ± 0.07 m; *p* < 0.001). In addition, men had higher weight compared to female patients (mean weight = 65 ± 13 versus 60 ± 14 kg; *p* = 0.009).

### Personal medical, surgical and family history of cancer

**Figure 1.**
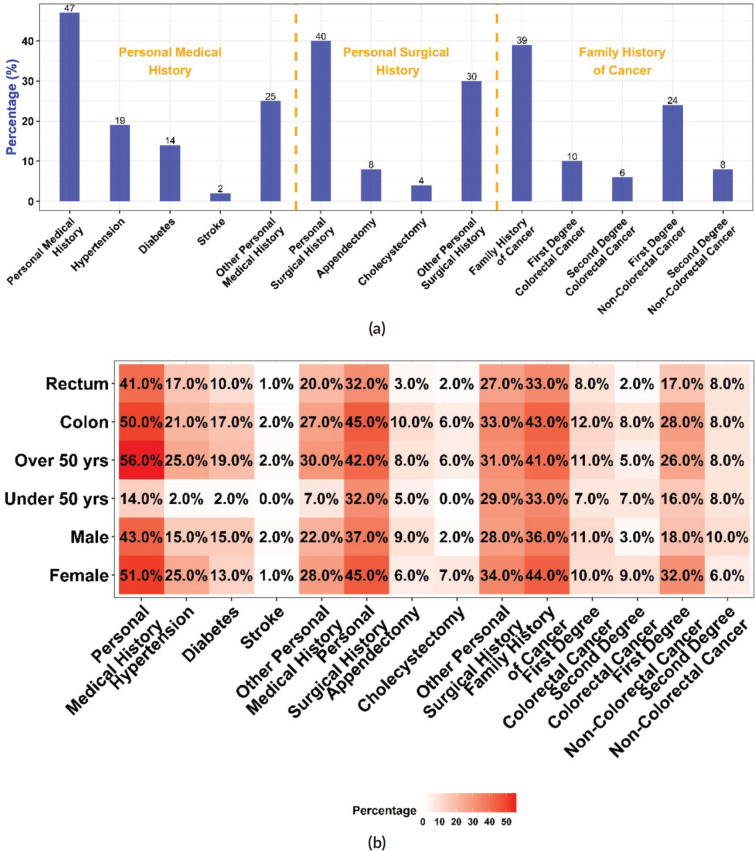
Personal medical, surgical and family history of cancer. (a): Proportions of CRC patients with pre-existing comorbidities, prior surgical history, and family history of cancer. (b): Proportions of CRC patients with personal, surgical, and family history of cancer stratified by CRC anatomic site, age group and sex.

All CRC patients’ personal medical, surgical and family histories were analysed. The results showed that 119 (47%) patients had a pre-existing medical condition in which hypertension was the most prevalent, manifesting in 49 (19%) of the patients, followed by diabetes, which was present in 36 (14%) of the studied population (Table 2 and Figure 1a). Patients with a positive personal medical history had a significantly higher mean age (64 ± 11 years) than those without (55 ± 13 years) (*p* < 0.001) and the risk increased by 7% for every additional year of age. (OR = 1.07; 95% CI = 1.05, 1.10; *p* < 0.001). In addition, they had a higher mean BMI (24.1 ± 4.8; *p* = 0.008), higher frequency of metastasis (39%; *p* = 0.045), and hepatic metastases (31%; *p* = 0.002). CRC patients with pre-existing onset of diabetes were older (65 ± 10; *p* < 0.001), had higher mean BMI (25.0 ± 5.1; *p* = 0.020), and had higher CA 19-9 levels (reference range ≥ 35 U/mL) compared to those without diabetes (50% versus 25%; *p* = 0.024). Moreover, CRC patients manifesting antecedent hypertension were older (68 ± 9 years; *p* < 0.001), experienced more incidents of stroke (6.1%; *p* = 0.024), had more cholecystectomies (14%; *p* = 0.001), and had higher CEA levels (≥ 5 ng/mL) (59%; *p* = 0.050).

Our study reported that 102 (40%) of CRC patients had undergone surgery. Of those who underwent surgery, 19 (19%) had an appendectomy and 11 (11%) underwent a cholecystectomy. Personal surgical history was more common in colon cancer patients 71 (45%) than in those with rectal cancer 31 (32%) (Figure 1b). This difference in surgical history between the two groups was statistically significant, with a value of *p* = 0.040. Patients with a personal surgical history were more likely to have colon than rectal cancer (OR = 1.74; 95% CI = 1.03, 2.97; *p* = 0.041) (Table 3 and Figure 1b). The same trend was noticed concerning appendectomy (*p* = 0.041), (OR = 3.47; 95% CI = 1.12, 15.2; *p* = 0.053). Patients with a positive surgical history had a significantly higher mean age (61 ± 13 years) compared to those without (58 ± 12 years) (*p* = 0.024), and the tumours were more localised in the colon (70%) than in the rectum (30%) (*p* = 0.040). The overweight and obesity categories showed a significant difference in CRC frequency based on surgical history (*p* = 0.023) and a significant difference in metastasis occurrence (40%; *p* = 0.036) and hepatic metastases (30%; *p* = 0.015). Patients who underwent appendectomy had more tumours localised in the colon than in the rectum (84% versus 16%; *p* = 0.041) and had lower CEA levels compared with those who did not undergo this type of surgery (10% versus 45%; *p* = 0.043).

A family history of cancer was present in 39% of CRC patients (Figure 1a). Of these patients, 24 (27%) reported a family history of CRC in one or more first-degree relatives, whereas 55 (61%) had one or multiple first-degree family members with other types of cancer. We noted a significantly higher prevalence of diabetes in CRC patients with a family history of cancer than in those with no such history (22%; *p* = 0.011).

### Histological classification, staging and metastasis

Adenocarcinomas accounted for 94% of all CRC cases, of these, 54% were well differentiated. The proportion of stageable adenocarcinomas was 66%. Out of the cases that could be classified, 34% were stage III (Table 4). Patients with colon cancer had a higher probability of being diagnosed at stage IV than those with rectal cancer (75% versus 25%; *p* < 0.001). Stage IV CRC patients had higher CEA levels (≥ 5 ng/mL) (80%) compared with patients in other stages of the disease (*p* = 0.002).

**Figure 2.**
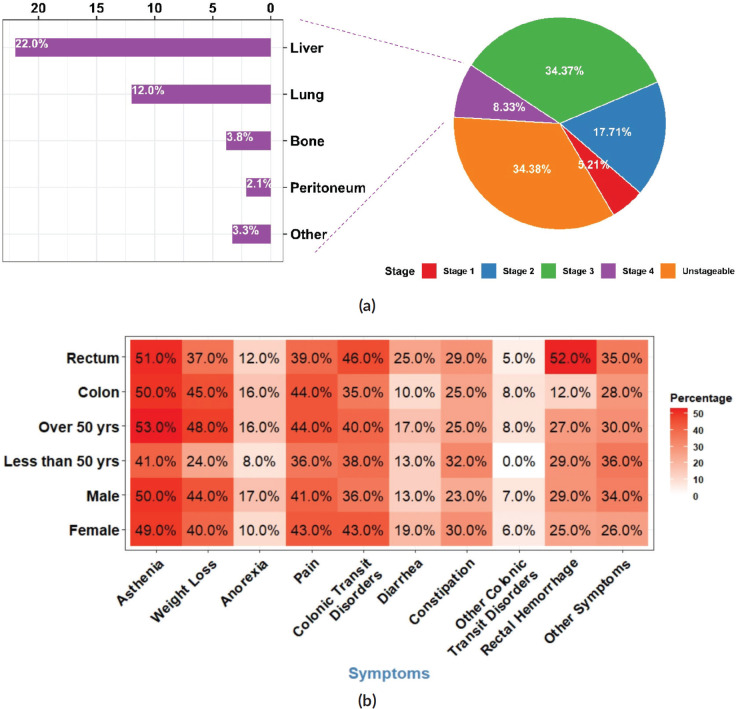
Staging, metastasis and symptoms (a): Distribution of CRC patients according to tumour stage at diagnosis with a proportional breakdown of metastatic status. (b): Heatmap illustrating the distribution of symptoms in CRC patients according to sex, age group and tumour anatomic site. Each tile represents the percentage of patients with a specific symptom.

Approximately one-third of the CRC patients in our study presented tumours with distant metastases. Hepatic and pulmonary metastases were the most frequent, accounting for 22% and 12% of all CRC cases in our study, respectively (Figure 2a). The difference between males and females regarding the presence of metastasis was significant (23% of females versus 39% of male patients; *p* = 0.010).

The occurrence of metastatic tumours was higher amongst male patients in comparison to females. The same trend was observed for liver and lung metastases (*p* = 0.031 and 0.040, respectively). Men developed hepatic and pulmonary metastases more often than women (OR = 2.03; 95% CI = 1.07, 3.99; *p* = 0.033) and (OR = 2.50; 95% CI = 1.07, 6.59; *p* = 0.045) respectively. CRC patients with metastasis presented higher CEA levels and lower CA 19-9 levels (74%; *p* < 0.001) and (46%; *p* < 0.001) respectively.

### Symptoms and treatment

The symptoms leading to CRC diagnosis were nonspecific. The most frequently observed signs at the time of diagnosis were asthenia (50%), weight loss (42%), pain (42%), and colonic transit disorders including diarrhoea and constipation (40%). Late-onset CRC patients experienced weight loss more frequently (*n* = 83, 48%) than early-onset patients (*n* = 12, 24%) (*p* = 0.002), while diarrhoea was more frequent in rectal cancer patients (*n* = 23, 25 %) than in colon cancer patients (*n* = 14, 9.7%) (*p* = 0.001) (Figure 2b).

Over 70% of diagnosed CRC cases received at least one type of treatment or a combination at the facility level. Of these patients, 85% received chemotherapy, 34% were treated with targeted therapy, 20% with radiotherapy, and 18% with radiochemotherapy. No discernible statistically significant differences in treatment accessibility were noted between the males and females. 83% of CRC patients who received chemotherapy had late-onset disease, 35% received it in combination with targeted therapy, and 22% with radiotherapy.

### Lifestyle behaviours and biomarkers

In the present study, information regarding KRAS mutation status was available for a cohort of 92 patients diagnosed with CRC (Table 5). Among these patients, 54 (59%) exhibited mutations in the KRAS gene, while the remaining 38 (41%) displayed a wild-type KRAS genotype. Similarly, data for 60 CRC patients regarding NRAS and BRAF mutation status were available showing that only 10% and 5% of these tumours presented mutations respectively.

## Discussion

In summary, 255 CRC patients were included in our study. Of these patients, approximately 57% were males, indicating a slight male predominance in our sample. This finding is consistent with the global incidence data of CRC, which indicates that 55% of cases are male [1].

Moreover, the proportion of colon cancers among all CRC cases in our study generally agreed with those recorded worldwide. Nearly two-thirds (62%) of patients had tumours localised in the colon, revealing a prevailing trend of colon localisation. In a study conducted in the Algerian East, 68.9% of CRC cases were found in the colon [19]. Similarly, 65% of CRC cases were localised in the colon in the USA, which records 8.4% of the global CRC incidence [20], and a national retrospective cohort study in England identified 63.5% of colon cancer [21], while in China, which contributes to 30% of the global CRC incidence, 55.1% of all CRC cases were colon cancers [1].

In our study, 78% of diagnosed CRC patients were aged 50 years or older. This indicates that CRC incidence remains more frequent in older patients in the study population, which corroborates the trend observed globally [20, 22]. The proportion of early-onset CRC patients was similar to that found in eastern Algeria (26.7%) [19] and Saudi Arabia (25%) [23], but higher than the results reported in the US (10%) [20]. This suggests that the incidence of CRC in younger adults may be increasing in our population and that there may be other factors contributing to CRC incidence, such as environmental or genetic factors. We noticed that early-onset patients (who were below the age of 50 years) were more likely to have primary rectal cancer than colon tumours when compared to late-onset patients (aged 50 years or older) which matches the findings reported in several countries like the USA and Japan [20, 24].

Over the last decade, the incidence of rectal cancer in the United States has gradually increased among those under the age of 50, with an annual increase of around 2% [25]. Similarly, findings from a European study [26] spanning from 1990 to 2016 show varying rates of increase in rectal cancer incidence across different age groups: a 3.5% annual rise among individuals aged 20–29 years, a 1.6% increase among those aged 30–39 years, and no significant change in trend among those aged 40–49 years. In contrast, China had a modest drop in rectal cancer incidence between 2005 and 2015, regardless of age at diagnosis, but was prominently expressed within the 35–49 age group seeing a 1.6% fall [27].

Algeria has experienced a significant shift in its disease burden, transitioning from a predominance of infectious diseases to the dominance of chronic non-communicable diseases (NCDs) as the leading cause of mortality. The country has made significant progress in improving living conditions and developing a free public health security system supported by the government [28]. While Algeria has an extensive and relatively comprehensive social security system, which includes several programs that target a wide range of vulnerable groups, there is room for all programs to improve in terms of coverage and adequacy of benefits [29]. The epidemiological transition has led to a high prevalence of NCDs, including diabetes, obesity, cardiovascular diseases and hypertension. According to the International Diabetes Federation [30], the relative prevalence of diabetes in Algeria was 7.1% in the age group of 20–79 years in 2021. Furthermore, according to Algeria’s STEPS survey from 2016 to 2017 [31], the rate of obesity ranges between 14.1% in men and 30.1% in women (21.8% for both sexes), and the proportion of high blood pressure was 31.6% [32].

Furthermore, in our study, the most frequent associated factors with CRC were hypertension, diabetes, appendectomy and family history of cancer. Compared to individuals with normotension, Zhang *et al* [8] found in a meta-analysis that individuals with hypertension had an increased CRC risk. The same trend was observed in a large study conducted by Kaneko *et al* [33], which revealed that elevated systolic and diastolic blood pressure levels were linked to a higher risk of incident CRC highlighting therefore the importance of supervising blood pressure to identify individuals at higher risk of developing CRC. Although the specific mechanisms underlying the link between hypertension and CRC remain unknown, these findings indicate a correlation that merits further investigation. Diabetes prevalence was associated with an increased risk of all CRC types [5, 8]. One reason that can explain this relationship is the shared risk factors between both conditions such as dietary and genetic factors. Another explanation could be that both conditions share molecular and cellular pathways, for instance, epithelial cell injury, inflammatory responses` activation and Wnt/β-catenin pathways [34].

The following studies attempted to investigate the correlation between a family history of all cancers and CRC risk yielding conflicting results. In a meta-analysis performed by Far *et al* [35], a positive first-degree family history of CRC showed an 87% statistically significant increase in CRC risk (RR = 1.87; 95% CI = 1.68, 2.09). As shown in a case-control study conducted by Negrichi and Taleb [36] in eastern Algeria, a family history of cancer in one of the first-degree relatives showed a significant relationship with the risk of developing CRC (OR = 2.46; 95% CI = 1.50, 4.05; *p* < 0.001), however, a more recent investigation by Lewandowska *et al* [6] did not find a significant association, which may indicate that other factors, such as genetic polymorphisms, lifestyle, and dietary habits, could also play a role in CRC predisposition. These contrasting findings suggest that the association between family history, which is already an established risk factor for CRC and included in several screening guidelines and risk prediction models, and the risk of CRC may be complex and requires further examination.

Several studies have investigated the relationship between appendectomy and CRC risk, with some showing an increased risk of CRC after appendectomy, while others have either found no association or a potential protective effect. In a meta-analysis, individuals who had experienced an appendectomy had an elevated risk of CRC, implying a possible link with CRC development [7]. Conversely, a 14 years of follow-up large retrospective cohort study found that appendectomy patients had a 1.14 times higher risk of developing CRC than the general population [37]. However, the increased risk was observed in another study, and it was primarily noticed in the first year after the appendectomy which may represent a potential bias. No statistically significant correlation was identified between appendectomy and the risk of CRC after adjusting for an appropriate delay period after surgery [38]. Recently, a study by Rothwell *et al* [39] reported an inverse relationship between undergoing appendectomy and the risk of colon cancer. Although there was a 9% reduction in CRC risk associated with appendectomy, it did not result in a lower overall CRC risk (HR = 0.91; 95% CI = 0.79, 1.02). The complex interplay between appendectomy, gut microbiota changes, inflammation, and CRC risk warrants further investigation to clarify the precise nature of this relationship and potential clinical implications.

Lifestyle changes, particularly in urban areas, have resulted in an unhealthy diet consisting of processed and high-fat foods. A Westernized diet, which is rich in red and processed meats, refined sugars, and saturated fats has replaced the traditional Algerian diet, rich in fruits, vegetables, and lean meats. This shift, combined with sedentary lifestyles and lack of physical activity, has been linked to an increased risk of CRC [36].

Furthermore, the Algerian National Cancer Screening Committee introduced CRC screening into the national cancer plan after the 2015–2019 program overlooked it. This decision followed the publication of results from the National Cancer Registry Network in January 2017, highlighting the significant incidence of CRC. Pilot studies using immunological tests were launched in three regions: Bejaia, Annaba and Batna. These tests aim to detect blood in the stool. Despite a modest overall participation rate of 26%, the rate rose to 98% among those with positive test results. A lack of awareness and screening programs has contributed to the late detection of CRC cases in Algeria. Unlike some Western countries, where routine screenings and awareness campaigns are common, Algeria lacks a comprehensive nationwide screening program. As a result, many cases are diagnosed at advanced stages, reducing the chances of successful treatment and survival [40, 41].

The current study results could significantly contribute to the development of a comprehensive approach to CRC prevention, diagnosis and treatment in Algeria. It offers insights into age-specific patterns, sex-based differences, and anatomic sites, which could enhance our understanding of CRC in this specific region. Public health interventions aimed at increasing awareness of CRC, supporting healthy dietary practices, and mitigating exposure to risk factors may benefit the population.

Certain limitations may affect the study’s results, including potential biases associated with retrospective data collection, the single-centre nature of the study, the small population number, and missing information in medical records for some variables which could potentially affect the generalisability of these findings.

## Conclusion

CRC incidence rates are increasing in Algeria. This retrospective study demonstrates the current state of CRC incidence in the northwest region of the country. Moreover, the study findings provide valuable data for healthcare planning and resource allocation which include ensuring access to screening services, diagnostic tools, treatment facilities, and supportive care for CRC patients to address the needs of the population.

## List of abbreviations

BMI, Body mass index; BRAF, B-Raf Proto-Oncogene, Serine/Threonine Kinase; CA 19-9, Carbohydrate antigen 19-9; CEA, carcinoembryonic antigen; CI, Confidence interval; CRC, colorectal cancer; GLOBOCAN, Global cancer observatory; HR, Hazard ratio; NCD, non-communicable disease; NRAS, Neuroblastoma; RAS; OR, odds ratio; RR, Relative risk.

## Conflicts of interest

The authors declare that they have no known competing financial interests or personal relationships that could have appeared to influence the work reported in this paper.

## Funding

This research did not receive any specific grant from funding agencies in the public, commercial, or not-for-profit sectors.

## Author contributions

SE conceptualisation, data curation, formal analysis, investigation, methodology, visualisation, writing – original draft, writing – review & editing. SM supervision, validation. DE conceptualisation, data curation, investigation, methodology. DY conceptualisation, methodology, project administration, resources, supervision, validation. MBK supervision, validation, writing – review & editing.

## Figures and Tables

**Table 1.** Patients’ demographic characteristics stratified by sex, tumour anatomic site and age category.

Characteristic	*N*	Sex			Anatomic site			Age category			Overall *N* = 255 ^a^
		Female *N* = 110 ^a^	Male *N* = 145 ^a^	*p*-value ^b^	Colon *N* = 158 ^a^	Rectum *N* = 97 ^a^	*p*-value ^b^	< 50 *N* = 56 ^a^	≥ 50 *N* = 199 ^a^	*p*-value ^b^	
Year	255			0.8			0.6			<0.001*	
2018		30 (27%)	38 (26%)		41 (26%)	27 (28%)		28 (50%)	40 (20%)		68 (27%)
2019		28 (25%)	31 (21%)		33 (21%)	26 (27%)		10 (18%)	49 (25%)		59 (23%)
2020		25 (23%)	37 (26%)		39 (25%)	23 (24%)		8 (14%)	54 (27%)		62 (24%)
2021		27 (25%)	39 (27%)		45 (28%)	21 (22%)		10 (18%)	56 (28%)		66 (26%)
Age	255			0.300			0.011*	-	-	-	
Mean ± SD		58 ± 13	60 ± 13		61 ± 13	57 ± 13		-	-		59 ± 13
Range		22, 89	20, 91		22, 91	20, 88		-	-		20, 91
Age groups	255			0.600				-	-		
[20–30]		2 (1.8%)	3 (2.1%)		3 (1.9%)	2 (2.1%)		-	-		5 (2.0%)
[30–40]		4 (3.6%)	8 (5.5%)		5 (3.2%)	7 (7.2%)		-	-		12 (4.7%)
[40–50]		21 (19%)	18 (12%)		18 (11%)	21 (22%)		-	-		39 (15%)
[50–60]		32 (29%)	40 (28%)		47 (30%)	25 (26%)		-	-		72 (28%)
[60–70]		31 (28%)	41 (28%)		45 (28%)	27 (28%)		-	-		72 (28%)
[70–80]		13 (12%)	28 (19%)		29 (18%)	12 (12%)		-	-		41 (16%)
[80–90]		7 (6.4%)	6 (4.1%)		10 (6.3%)	3 (3.1%)		-	-		13 (5.1%)
[90–100]		0 (0%)	1 (0.7%)		1 (0.6%)	0 (0%)		-	-		1 (0.4%)
Age category	255			0.400			0.007*	-	-	-	
< 50		27 (25%)	29 (20%)		26 (16%)	30 (31%)		-	-		56 (22%)
≥ 50		83 (75%)	116 (80%)		132 (84%)	67 (69%)		-	-		199 (78%)
Anatomic site	255			0.500	-	-	-			0.007*	
Colon		71 (65%)	87 (60%)		-	-		26 (46%)	132 (66%)		158 (62%)
Rectum		39 (35%)	58 (40%)		-	-		30 (54%)	67 (34%)		97 (38%)
Sex	255	-	-	-			0.500			0.400	
Female		-	-		71 (45%)	39 (40%)		27 (48%)	83 (42%)		110 (43%)
Male		-	-		87 (55%)	58 (60%)		29 (52%)	116 (58%)		145 (57%)
Patients’ weights	155			0.009*			> 0.900			0.300	
Mean ± SD		60 ± 14	65 ± 13		63 ± 14	63 ± 13		60 ± 13	63 ± 14		63 ± 13
Range		37, 104	43, 98		38, 98	37, 104		41, 84	37, 104		37, 104
Patients’ heights	141			< 0.001*			0.700			0.030	
Mean ± SD		1.58 ± 0.07	1.71 ± 0.08		1.66 ± 0.10	1.66 ± 0.11		1.70 ± 0.09	1.65 ± 0.10		1.66 ± 0.10
Range		1.46, 1.80	1.46, 1.97		1.46, 1.97	1.46, 1.85		1.55, 1.85	1.46, 1.97		1.46, 1.97
BMI	140			0.110			0.400			0.019*	
Mean ± SD		24.1 ± 5.8	22.4 ± 3.6		23.2 ± 4.5	22.9 ± 4.9		21.2 ± 4.5	23.4 ± 4.6		23.1 ± 4.7
Range		11.7, 39.1	15.4, 33.7		15.2, 34.4	11.7, 39.1		15.2, 32.1	11.7, 39.1		11.7, 39.1
BMI category	140			0.026*			0.300			0.200	
Underweight		9 (16%)	12 (14%)		14 (17%)	7 (13%)		6 (29%)	15 (13%)		21 (15%)
Normal Weight		24 (43%)	52 (62%)		25 (30%)	11 (20%)		11 (52%)	65 (55%)		76 (54%)
Overweight		17 (30%)	19 (23%)		5 (6.0%)	2 (3.6%)		3 (14%)	33 (28%)		36 (26%)
Obesity		6 (11%)	1 (1.2%)		40 (48%)	36 (64%)		1 (4.8%)	6 (5.0%)		7 (5.0%)
Socioeconomic Level	72			0.031*			0.200			0.024*	
High		6 (19%)	10 (24%)		12 (27%)	4 (15%)		0 (0%)	16 (28%)		16 (22%)
Middle class		13 (42%)	26 (63%)		12 (27%)	5 (19%)		8 (57%)	31 (53%)		39 (54%)
Low		12 (39%)	5 (12%)		21 (47%)	18 (67%)		6 (43%)	11 (19%)		17 (24%)
Higher educational status	63			0.014*			0.110			> 0.900	
Secondary or higher education		8 (33%)	16 (41%)		12 (32%)	8 (32%)		3 (30%)	21 (40%)		24 (38%)
Middle school		4 (17%)	16 (41%)		0 (0%)	1 (4.0%)		4 (40%)	16 (30%)		20 (32%)
Primary education		12 (50%)	6 (15%)		8 (21%)	10 (40%)		3 (30%)	15 (28%)		18 (29%)
Never schooled		0 (0%)	1 (2.6%)		18 (47%)	6 (24%)		0 (0%)	1 (1.9%)		1 (1.6%)

* *p*-value significant at below 0.050 threshold;

a *n* (%);

b Wilcoxon rank sum test;

Fisher’s exact test; Pearson’s chi-squared test

**Table 2.** Age, sex, and anatomical-related differences of personal medical, surgical and family history of cancer.

Characteristic	*N*	Sex			Anatomic site			Age category			Overall *N* = 255 ^a^
		Female *N* = 110 ^a^	Male *N* = 145 ^a^	*p*-value ^b^	Colon *N* = 158 ^a^	Rectum *N* = 97 ^a^	*p*-value ^b^	< 50 *N* = 56 ^a^	≥ 50 *N* = 199 ^a^	*p*-value ^b^	
Personal medical history	255	56 (51%)	63 (43%)	0.200	79 (50%)	40 (41%)	0.200	8 (14%)	111 (56%)	< 0.001*	119 (47%)
Hypertension	253	27 (25%)	22 (15%)	0.058	33 (21%)	16 (17%)	0.400	1 (1.8%)	48 (24%)	< 0.001*	49 (19%)
Diabetes	253	14 (13%)	22 (15%)	0.600	27 (17%)	9 (9.5%)	0.093	1 (1.8%)	35 (18%)	0.003*	36 (14%)
Stroke	253	1 (0.9%)	3 (2.1%)	0.600	3 (1.9%)	1 (1.1%)	> 0.900	0 (0%)	4 (2.0%)	0.600	4 (1.6%)
Other personal medical history	252	30 (28%)	32 (22%)	0.300	43 (27%)	19 (20%)	0.200	4 (7.1%)	58 (30%)	< 0.001*	62 (25%)
Personal surgical history	255	49 (45%)	53 (37%)	0.200	71 (45%)	31 (32%)	0.040*	18 (32%)	84 (42%)	0.200	102 (40%)
Appendicectomy	250	7 (6.4%)	12 (8.5%)	0.500	16 (10.3%)	3 (3.2%)	0.041*	3 (5.4%)	16 (8.2%)	0.600	19 (7.6%)
Cholecystectomy	250	8 (7.3%)	3 (2.1%)	0.062	9 (5.8%)	2 (2.1%)	0.200	0 (0%)	11 (5.7%)	0.130	11 (4.4%)
Other personal surgical history	250	37 (34%)	39 (28%)	0.300	51 (33%)	25 (27%)	0.300	16 (29%)	60 (31%)	0.700	76 (30%)
Family history of cancer	230	44 (44%)	46 (36%)	0.200	62 (43%)	28 (33%)	0.140	17 (33%)	73 (41%)	0.300	90 (39%)
First degree CRC	232			0.800			0.700			0.700	
One		9 (8.8%)	11 (8.5%)		14 (9.6%)	6 (7.0%)		3 (5.8%)	17 (9.4%)		20 (8.6%)
multiple		1 (1.0%)	3 (2.3%)		3 (2.1%)	1 (1.2%)		1 (1.9%)	3 (1.7%)		4 (1.7%)
No history		92 (90%)	116 (89%)		129 (88%)	79 (92%)		48 (92%)	160 (89%)		208 (90%)
Second degree CRC	232			0.200			0.300			0.600	
One		6 (5.8%)	3 (2.3%)		8 (5.5%)	1 (1.1%)		3 (5.8%)	6 (3.3%)		9 (3.9%)
Multiple		3 (2.9%)	1 (0.8%)		3 (2.1%)	1 (1.1%)		1 (1.9%)	3 (1.7%)		4 (1.7%)
No history		94 (91%)	125 (97%)		134 (92%)	85 (98%)		48 (92%)	171 (95%)		219 (94%)
First degree NO- CRC	229			0.054			0.200			0.200	
One		27 (27%)	20 (16%)		33 (23%)	14 (16%)		6 (12%)	41 (23%)		47 (21%)
Multiple		5 (5.0%)	3 (2.3%)		7 (4.9%)	1 (1.2%)		2 (3.8%)	6 (3.4%)		8 (3.5%)
No history		69 (68%)	105 (82%)		104 (72%)	70 (82%)		44 (85%)	130 (73%)		174 (76%)
Second degree non- CRC	231			0.080			0.700			0.130	
One		0 (0%)	6 (4.7%)		3 (2.1%)	3 (3.4%)		3 (5.8%)	3 (1.7%)		6 (2.6%)
Multiple		6 (5.9%)	7 (5.4%)		9 (6.3%)	4 (4.6%)		1 (1.9%)	12 (6.7%)		13 (5.6%)
No history		96 (94%)	116 (90%)		132 (92%)	80 (92%)		48 (92%)	164 (92%)		212 (92%)

* *p*-value significant at below 0.050 threshold;

a *n* (%);

b Wilcoxon rank sum test;

Fisher’s exact test; Pearson’s chi-squared test

**Table 3.** Association of clinical characteristics with the risk of CRC according to CRC anatomic site and sex.

Variable	Colon, *N* = 158 ^a^	Rectum, *N* = 97 ^a^	OR ^b^	95% CI ^b^	*p*-value
Age category					
<50	26 (16%)	30 (31%)	—	—	
≥50	132 (84%)	67 (69%)	2.27	1.25, 4.17	0.007*
Personal surgical history	**Colon, *N* = 158 ^a^**	**Rectum, *N* = 97 ^a^**			
No	87 (55%)	66 (68%)	—	—	
Yes	71 (45%)	31 (32%)	1.74	1.03, 2.97	0.041*
Appendectomy	**Colon, *N* = 156 ^a^**	**Rectum, *N* = 94 ^a^**			
No	140 (89.7)	91 (96.8%)	—	—	
Yes	16 (10.3%)	3 (3.2%)	3.47	1.12, 15.2	0.053
Stage	**Colon, *N* = 121 ^a^**	**Rectum, *N* = 71 ^a^**			
1	8 (6.6%)	2 (2.8%)	—	—	
2	32 (26%)	2 (2.8%)	4.00	0.43, 37.9	0.200
3	32 (26%)	34 (48%)	0.24	0.03, 1.02	0.081
4	12 (9.9%)	4 (5.6%)	0.75	0.09, 4.86	0.800
Unstageable	37 (31%)	29 (41%)	0.32	0.05, 1.39	0.200
**Variable**			**OR ^b^**	**95% CI ^b^**	***p*-value**
Metastasis	**Female, *N* = 103 ^a^**	**Male, *N* = 136 ^a^**			
No	79 (76.7%)	83 (61%)	—	—	
Yes	24 (23.3%)	53 (39%)	2.10	1.20, 3.77	0.011*
Hepatic metastases					
No	87 (84.5%)	99 (72.8%)	—	—	
Yes	16 (15.5%)	37 (27.1%)	2.03	1.07, 3.99	0.033*
Pulmonary metastases					
No	96 (93.2%)	115 (84.6%)	—	—	
Yes	7 (6.8%)	21 (15.4%)	2.50	1.07, 6.59	0.045*

* *p*-value statistically significant at below 0.050 threshold;

a *n* (%)

Abbreviations: OR, Odds Ratio; CI, Confidence Interval

**Table 4.** Histological classification, staging, and metastasis status according to sex, age and anatomic site.

Characteristic	*N*	Sex			Anatomic site			Age category			Overall *N* = 255 ^a^
		Female *N* = 110 ^a^	Male *N* = 145 ^a^	*p*-value ^b^	Colon *N* = 158 ^a^	Rectum *N* = 97 ^a^	*p*-value ^b^	< 50 *N* = 56 ^a^	≥ 50 *N* = 199 ^a^	*p*-value ^b^	
Adenocarcinoma	255	102 (93%)	137 (94%)	0.600	149 (94%)	90 (93%)		54 (96%)	185 (93%)	0.600	239 (94%)
Degree of differentiation of adenocarcinoma	220			> 0.900			0.500			> 0.900	
Well-differentiated		51 (54%)	62 (50%)		65 (48%)	48 (57%)		26 (57%)	87 (50%)		113 (51%)
Moderately differentiated		34 (36%)	48 (38%)		56 (41%)	26 (31%)		16 (35%)	66 (38%)		82 (37%)
Well to moderately differentiated		2 (2.1%)	3 (2.4%)		4 (2.9%)	1 (1.2%)		1 (2.2%)	4 (2.3%)		5 (2.3%)
Poorly differentiated		3 (3.2%)	6 (4.8%)		5 (3.7%)	4 (4.8%)		1 (2.2%)	8 (4.6%)		9 (4.1%)
Not applicable		5 (5.3%)	6 (4.8%)		6 (4.4%)	5 (6.0%)		2 (4.3%)	9 (5.2%)		11 (5.0%)
Stage	192			> 0.900			< 0.001*			0.500	
1		5 (5.8%)	5 (4.7%)		8 (6.6%)	2 (2.8%)		2 (5.3%)	8 (5.2%)		10 (5.2%)
2		16 (19%)	18 (17%)		32 (26%)	2 (2.8%)		5 (13%)	29 (19%)		34 (18%)
3		29 (34%)	37 (35%)		32 (26%)	34 (48%)		11 (29%)	55 (36%)		66 (34%)
4		7 (8.1%)	9 (8.5%)		12 (9.9%)	4 (5.6%)		2 (5.3%)	14 (9.1%)		16 (8.3%)
Unstageable		29 (34%)	37 (35%)		37 (31%)	29 (41%)		18 (47%)	48 (31%)		66 (34%)
*T* (Tumour)	192			0.077			0.032*			0.500	
1		0 (0%)	1 (0.9%)		1 (0.8%)	0 (0%)		0 (0%)	1 (0.6%)		1 (0.5%)
2		11 (13%)	9 (8.5%)		16 (13%)	4 (5.6%)		4 (11%)	16 (10%)		20 (10%)
3		51 (59%)	79 (75%)		85 (70%)	45 (63%)		25 (66%)	105 (68%)		130 (68%)
4		23 (27%)	17 (16%)		19 (16%)	21 (30%)		8 (21%)	32 (21%)		40 (21%)
x		1 (1.2%)	0 (0%)		0 (0%)	1 (1.4%)		1 (2.6%)	0 (0%)		1 (0.5%)
*N* (Node)	192			0.300			< 0.001*			> 0.900	
0		33 (38%)	34 (32%)		60 (50%)	7 (9.9%)		14 (37%)	53 (34%)		67 (35%)
1		27 (31%)	29 (27%)		32 (26%)	24 (34%)		12 (32%)	44 (29%)		56 (29%)
2		18 (20.5%)	22 (20.9%)		21 (17.5%)	19 (26.4%)		6 (16%)	34 (21.6%)		40 (21%)
3		0 (0%)	1 (0.9%)		0 (0%)	1 (1.4%)		0 (0%)	1 (0.6%)		1 (0.5%)
x		8 (9.3%)	20 (19%)		8 (6.6%)	20 (28%)		6 (16%)	22 (14%)		28 (15%)
*M* (Metastasis)	183			0.300			0.600			0.140	
0		53 (64%)	64 (64%)		71 (62%)	46 (68%)		22 (61%)	95 (65%)		117 (64%)
1		8 (9.6%)	16 (16%)		15 (13%)	9 (13%)		2 (5.6%)	22 (15%)		24 (13%)
x		22 (27%)	20 (20%)		29 (25%)	13 (19%)		12 (33%)	30 (20%)		42 (23%)
Metastasis	239	24 (23.3%)	53 (39%)	0.010*	50 (33%)	27 (30%)	0.600	16 (29%)	61 (33%)	0.600	77 (32%)
Hepatic	239	16 (15.5%)	37 (27.1%)	0.031*	36 (24%)	17 (19%)	0.400	10 (18%)	43 (23%)	0.400	53 (22%)
Pulmonary	239	7 (6.8%)	21 (15.4%)	0.040*	13 (8.7%)	15 (17%)	0.057	3 (5.5%)	25 (14%)	0.100	28 (12%)
Peritoneal	240	2 (1.9%)	3 (2.2%)	> 0.900	4 (2.7%)	1 (1.1%)	0.700	0 (0%)	5 (2.7%)	0.600	5 (2.1%)
Bone	240	1 (1.0%)	8 (5.8%)	0.082	5 (3.3%)	4 (4.5%)	0.700	2 (3.6%)	7 (3.8%)	> 0.900	9 (3.8%)
Lymph node	239	0 (0%)	3 (2.2%)	0.300	2 (1.3%)	1 (1.1%)	> 0.900	1 (1.8%)	2 (1.1%)	0.500	3 (1.3%)

* *p*-value significant at below 0.050 threshold;

a *n* (%);

b Wilcoxon rank sum test;

Fisher’s exact test; Pearson’s chi-squared test

**Table 5.** Biomarkers and lifestyle behaviours stratified by sex, tumour anatomic site, and age category.

Characteristic	*N*	Sex			Anatomic site			Age category			Overall *N* = 255 ^a^
		Female *N* = 110 ^a^	Male *N* = 145 ^a^	*p*-value ^b^	Colon *N* = 158 ^a^	Rectum *N* = 97 ^a^	*p*-value ^b^	< 50 *N* = 56 ^a^	≥ 50 *N* = 199 ^a^	*p*-value ^b^	
KRAS status	92			0.600			0.700			0.700	
Mutated		26 (62%)	28 (56%)		35 (60%)	19 (56%)		15 (56%)	39 (60%)		54 (59%)
Wild type		16 (38%)	22 (44%)		23 (40%)	15 (44%)		12 (44%)	26 (40%)		38 (41%)
NRAS status	60			> 0.900			> 0.900			0.700	
Mutated		3 (10%)	3 (10%)		4 (10%)	2 (10%)		3 (14%)	3 (7.7%)		6 (10%)
Wild type		27 (90%)	27 (90%)		36 (90%)	18 (90%)		18 (86%)	36 (92%)		54 (90%)
BRAF status	60			0.200			0.500			> 0.900	
Mutated		3 (10%)	0 (0%)		3 (7.3%)	0 (0%)		1 (4.5%)	2 (5.3%)		3 (5.0%)
Wild type		27 (90%)	30 (100%)		38 (93%)	19 (100%)		21 (95%)	36 (95%)		57 (95%)
CEA	136			0.800			0.035*			0.300	
≤ 5		32 (56%)	46 (58%)		59 (63%)	19 (44%)		11 (48%)	67 (59%)		78 (57%)
≥ 5		25 (44%)	33 (42%)		34 (37%)	24 (56%)		12 (52%)	46 (41%)		58 (43%)
CEA level	136			0.900			0.045*			0.700	
Mean ± SD		76 ± 329	55 ± 151		65 ± 267	60 ± 177		10 ± 11	75 ± 264		64 ± 241
Range		0, 2,296	0, 1,000		0, 2,296	0, 1,000		0, 37	0, 2,296		0, 2,296
CA 19-9	135			0.800			0.900			0.200	
≤ 35		39 (70%)	57 (72%)		65 (71%)	31 (72%)		19 (83%)	77 (69%)		96 (71%)
≥ 35		17 (30%)	22 (28%)		27 (29%)	12 (28%)		4 (17%)	35 (31%)		39 (29%)
CA 19-9 level	135			0.300			> 0.900			0.200	
Mean ± SD		153 ± 337	96 ± 235		127 ± 288	106 ± 272		64 ± 207	131 ± 295		120 ± 282
Range		1, 1,200	1, 1,000		1, 1,000	1, 1,200		1, 1,000	1, 1,200		1, 1,200
Smoking	62	0 (0%)	46 (79%)	0.003*	29 (74%)	17 (74%)	> 0.900	4 (80%)	42 (74%)	> 0.900	46 (74%)
Alcohol	41	0 (0%)	15 (38%)	0.500	10 (43%)	5 (28%)	0.300	2 (67%)	13 (34%)	0.500	15 (37%)

* *p*-value significant at below 0.050 threshold;

a *n* (%);

b Wilcoxon rank sum test;

Fisher’s exact test; Pearson’s chi-squared test
